# Etymologia: Rotavirus

**DOI:** 10.3201/eid1708.ET1708

**Published:** 2011-08

**Authors:** Nancy Männikkö

**Affiliations:** Author affiliation: Centers for Disease Control and Prevention, Atlanta, GA, USA

**Keywords:** rotavirus, etymology, electron microscope, viruses

##  [ro′tə-vi′′rəs]

From the Latin *rota,* wheel, plus *virus*. After viewing the virus through an electron microscope in 1974, Flewett et al. suggested the name rotavirus on the basis of the pathogen’s shape. The International Committee on Taxonomy of Viruses approved the name 4 years later.

**Sources:** Dorland’s illustrated medical dictionary. 31st edition. Philadelphia: Saunders, 2007; Flewett TH, Bryden AS, Davies H, Woode GN, Bridger JC, Derrick JM. Relation between viruses from acute gastroenteritis of children and newborn calves. Lancet. 1974;304:61–3.PubMed ; Matthews RE. Third report of the International Committee on Taxonomy of Viruses. Classification and nomenclature of viruses. Intervirology. 1979;12:129–296.PubMed

